# The challenge of managing yellow rust (*Puccinia striiformis* f.sp. *tritici*) in winter wheat: how combined climate and pathogen stressors impact variability in genotype reactions

**DOI:** 10.3389/fpls.2023.1270087

**Published:** 2023-10-20

**Authors:** Radivoje Jevtić, Vesna Župunski

**Affiliations:** Laboratory for Phytopathology, Small Grains Department, Institute of Field and Vegetable Crops, Novi Sad, Serbia

**Keywords:** yellow rust, susceptibility, resistance, wheat, climatic factors

## Abstract

Despite the ongoing evolution of wheat pathogens due to the selection pressures of agro-ecological conditions, many studies have often overlooked the combined impact of both biotic and abiotic factors on disease occurrence. From 2016 to 2023, a comprehensive screening of obligate pathogens, including *B. graminis* f. sp. *tritici, P. graminis* f. sp. *tritici*, *P. triticina*, and *P. striiformis* f. sp. *tritici*, was carried out. This screening was conducted on a phenotyping platform encompassing 2715 winter wheat genotypes and their wild relatives, both with and without resistant genes (Lr, Yr, and Sr) for rust diseases. The data were analyzed using PCAmix, best subsets regression, and linear regression modeling. The findings from this study reveal that the plant reactions to leaf and yellow rust infections is far from straightforward. It is heavily influenced not only by prevalent rust races and climatic factors that impact pathogen life cycles but also by variations in the susceptibility reactions of wheat genotypes to the broader agro-ecological conditions. We also observed a tendency for leaf rust and yellow rust to coexist within the same host plant, even though yellow rust is typically considered more aggressive. We reported for the first time genes related to yellow rust resistance breakdown in Serbia in 2023. Lastly, we underscored the importance of investigating resistance responses to rust diseases not exclusively through the interrelation between resistance genes and pathogen virulence, but also by considering how plants respond to the combined stresses of abiotic and biotic factors. Consequently, our study sets the groundwork for further research into how plants respond to multiple stressors and contributes for further investigations related with effective integrated rust management.

## 1 Introduction

Pest control in wheat production is challenging due to the continuous changes in wheat pathogens under selection pressure from resistant varieties, applied pesticides, and changing climatic conditions. The estimation suggests that as much as 40% of the global food supply is currently lost to pests, with the potential for further damage heightened by shifts in temperature, precipitation patterns, and the rise in extreme weather events, as highlighted by Heeb et al. (2019). The most important obligate pathogens of wheat are the causal agents of rust diseases (*Puccinia graminis* f. sp. *tritici*, *Puccinia triticina*, *Puccinia striiformis* f. sp. *tritici*) and powdery mildew (*Blumeria graminis* f. sp. *tritici*). Yield loss of rust diseases could reach 70% (Junk et al., 2016), while stem rust was also reported to cause yield losses of up to 100% (Afzal et al., 2007). Despite the continuous changes in wheat pathogens under the selection pressures of agro-ecological conditions, the impact of the combined effects of biotic and abiotic factors on disease occurrence has often been neglected in studies (White et al., 2011; Juroszek and von Tiedemann, 2013). Heeb et al. (2019) promoted a strategy for climate-smart pest management (CSPM) but also noted that developing a general model for predicting climate change-induced pest outbreaks on a local scale in the short term is highly unlikely.

*Puccinia striiformis* f.sp. *tritici* is a pathogen with the ability to spread over long distances through air currents, which is also its primary mode of transmission (Zadoks, 1961; Hovmøller et al., 2011). Although uredospores can be observed on the host plant’s ears at high infection intensities, yellow rust is not transmitted through seeds. Telia production begins with the sexual stage of rust diseases in the late growing season, and teliospores from telia further infect alternative hosts. Pycniospores and aeciospores are formed on alternative hosts, and the life cycle is completed when aeciospores infect the host plants of wheat (Chen, 2005). During ideal conditions, 10 days are enough for yellow rust to complete its cycle from infection to the production of new spores. The disease cycle may repeat many times in one season.

There are six main genetic groups of yellow rust, each predominant in different regions worldwide: G1 in China; G2 in Nepal; G3 in Pakistan; G4 in the Middle East and Eastern Africa; G5 in the Mediterranean and Central Asia; and G6 in Northwestern Europe (Ali et al., 2014). The center of yellow rust diversity is the Himalayan region, where the greatest genetic changes occur, unlike the clonal population structure that dominates in Europe, America, and Australia (Wellings and Mcintosh, 1990; Hovmøller et al., 2011; Ali et al., 2014). Prior to 2011, the population of *P. striiformis* in Europe was predominantly clonal and depended on mutations with little influence from sexual recombination (Hovmøller et al., 2002). In a clonal population, mutations and subsequent selection would generate new virulent races against existing host resistance genes (Linde et al., 2002; de Vallavieille-Pope et al., 2012). The majority of clonal races were typical for the northwestern European genetic group, while exotic races had only a minor impact on wheat production (de Vallavieille-Pope et al., 2012). However, in 2011, two new races were discovered in many European countries, named Warrior and Kranich, which exhibited significantly greater genetic variability compared to the races of the previous clonal populations (Hovmøller et al., 2016). Warrior and Kranich caused immense problems in wheat production because of virulence against varieties carrying durable resistance to prevalent races of the yellow rust pathogen (Sørensen et al., 2014). These results emphasize the fact that, despite the continuous development of wheat varieties with resistance to the prevalent pathogen population, the introduction of new races of yellow rust can initiate infections of epidemic proportions, even at a continental level (Brown and Hovmøller, 2002).

Prevalent races (virulence phenotype) of yellow rust are grouped into genetic lineages and named Pst, followed by a sequential digit (Ali et al., 2014; Hovmøller et al., 2016; Thach et al., 2016). PstS1 is closely related with PstS2 and predominates in North America. PstS2 is predominant in East Africa (Hovmøller et al., 2008; Ali et al., 2014; Walter et al., 2016). PstS3 is prevalent in West Asia, southern Europe and North Africa (Ali et al., 2014). PstS4 consists of races prevalent on triticale in Northern Europe (Hovmøller et al., 2008; Hovmøller et al., 2016). Two races with specific microsatellite profiles belong to PstS5 (Ali et al., 2014; Hovmøller et al., 2016; Thach et al., 2016). PstS6 is a lineage prevalent in East Africa (Ali et al., 2014; Hovmøller et al., 2016; Thach et al., 2016). Yellow rust races belonging to lineages PstS7 (Warrior), PstS8 (Kranich), and PstS10 became prevalent in Europe from 2011, covering more than 80% of the investigated isolates (Ali et al., 2014). PstS10, formerly known as Warrior(-), became predominant in most parts of Europe since 2014 (Ali et al., 2014). Yellow rust races in the NW-European population detected before 2011 were part of a single clonal lineage termed PstS0 (Ali et al., 2014). PstS9 is related with PstS5 and was associated with epidemics in Central Asia since 2013 (Ali et al., 2014).

The presence of yellow rust in Serbia was first recorded in the genetic collection at Rimski šančevi by Jevtić et al. as early as 1997, and the first warning about the increased threat of its occurrence due to climate change was given by Jevtić and Jasnić in 2007 (Jevtić et al., 2023). Until 2014, the predominant rust species in Serbia was *Puccinia triticina*, which, in certain years (2001, 2004, and 2007), resulted in yield losses of up to 50% in experimental fields. However, in the 2013/2014 production season, *Puccinia striiformis* f.sp. *tritici* predominated over leaf rust and similarly, as in the rest of European countries, threatened wheat production (Jevtić et al., 2017; Jevtić et al., 2020). In 2014, winter temperatures in January (4.2°C) and February (6.1°C) exceeded the average temperatures (-0.1°C in January and 1.8°C in February) since 1964, and yellow rust prevailed over leaf rust and caused enormous damage in wheat production (Jevtić et al., 2020). During 2014, the disease indices of yellow rust ranged from 40% to 60% in wheat production areas (Jevtić et al., 2020). The outbreak of yellow rust in Serbia was caused by Warrior races (Jevtić et al., 2017; Jevtić et al., 2020).

Considering the mode of transmission of the yellow rust pathogen and its high ability to overcome host plant resistance, it was hypothesized in this study that the occurrence of rust diseases cannot be explained only by the influence of climatic factors on the life cycle of pathogens and the effectiveness of resistance genes. Rather, it can also be attributed to the overall genotype reaction to combined abiotic and biotic stressors. Consequently, the aim of this study was to examine factors affecting variability in the predominance of yellow over leaf rusts and to highlight fundamental issues in the control of rust diseases.

## 2 Materials and methods

### 2.1 Plant material and field trials

The screening of obligate pathogens (*B. graminis* f. sp. *tritici*, *P. graminis* f. sp. *tritici*, *P. triticina*, *P. striiformis* f. sp. *tritici*) in the period 2016-2023 was conducted on a phenotyping platform comprising 2715 winter wheat genotypes and wild relatives (**Supplementary Table 1**). Winter wheat genotypes were characterized with diverse genetic backgrounds and included susceptible as well as genotypes carrying resistant genes for rust diseases (Lr, Yr, and Sr).

The phenotyping platform was established every year in the locality of Rimski šančevi (Vojvodina, the northern province of Serbia) according to the methodology recommended by CIMMYT (1979) in the “Instructions for the management and reporting of results for wheat program international yield and screening nurseries”. The phenotyping platform is designed for the rapid assessment of a large number of advanced generation (F3-F7) lines of wheat genotypes under a wide range of climatic and disease conditions. Phenotyping was conducted under the direction of the Institute of Field and Vegetable Crops, Serbia (Laboratory for Phytopathology of the Small Grains Department) as a part of pre-breeding and breeding efforts in developing and promoting wheat lines with resistance to rust diseases and powdery mildew.

In the phenotyping platform, each genotype was sown in 1-m rows in five replicates with a row spacing of 20 cm, giving a plot area of 1 m^2^. The establishment of the phenotyping platform was in accordance with the methodology recommended by CIMMYT, which indicated that each genotype should be sown in one unreplicated 5-m row or using smaller row lengths but with replicates so that the sum of all row lengths is equal to 5 m. Additionally, field trials were set up under naturally occurring inoculum, and the soft wheat variety Barbee (*Triticum aestivum* ssp. *compactum*), known for its susceptibility to *Blumeria* and *Puccinia*, was used to control pathogen pressure on all tested genotypes. The optimal time for winter wheat sowing in the agro-ecological conditions in Serbia is October, and the mean sowing date for genotypes in phenotyping platform in the eight-year period was 20 October.

### 2.2 Disease assessments

Assessments of obligate pathogens were made using disease indices (DIs) at the growth stage 71-73 BBCH (kernel watery; early milk) known for its high association with yield (Wegulo et al., 2009). The DIs (%) were calculated as follows: DI (%) = [sum (class frequency × score of rating class)]/[(total number of plants) × (maximal score of rating class)] × 100. Consequently, DI is a product of disease incidence (mean percentage of plants infected per plot) and average disease severity defined as the percentage of relevant host tissues or organs covered by symptoms (Seem, 1984). Disease severity was determined using the modified Cobb’s scale (Peterson et al., 1948). The genotype reaction to yellow rust (GR YR) and leaf rust (GR LR) was categorized using the following scale: DI < 10% (GR=1) – resistant; 11 < DI < 20% (GR=2) – moderately resistant; 21 < DI < 30% (GR=3) – moderately susceptible; 31 < DI < 40% (GR=4) – moderately susceptible; 41 < DI < 50% (GR=5) – susceptible; 51 < DI < 60% – susceptible (GR=6); DI > 61 (GR=7) – susceptible.

### 2.3 Assessment of climatic factors in the phenotyping platform

**Figure 1:**
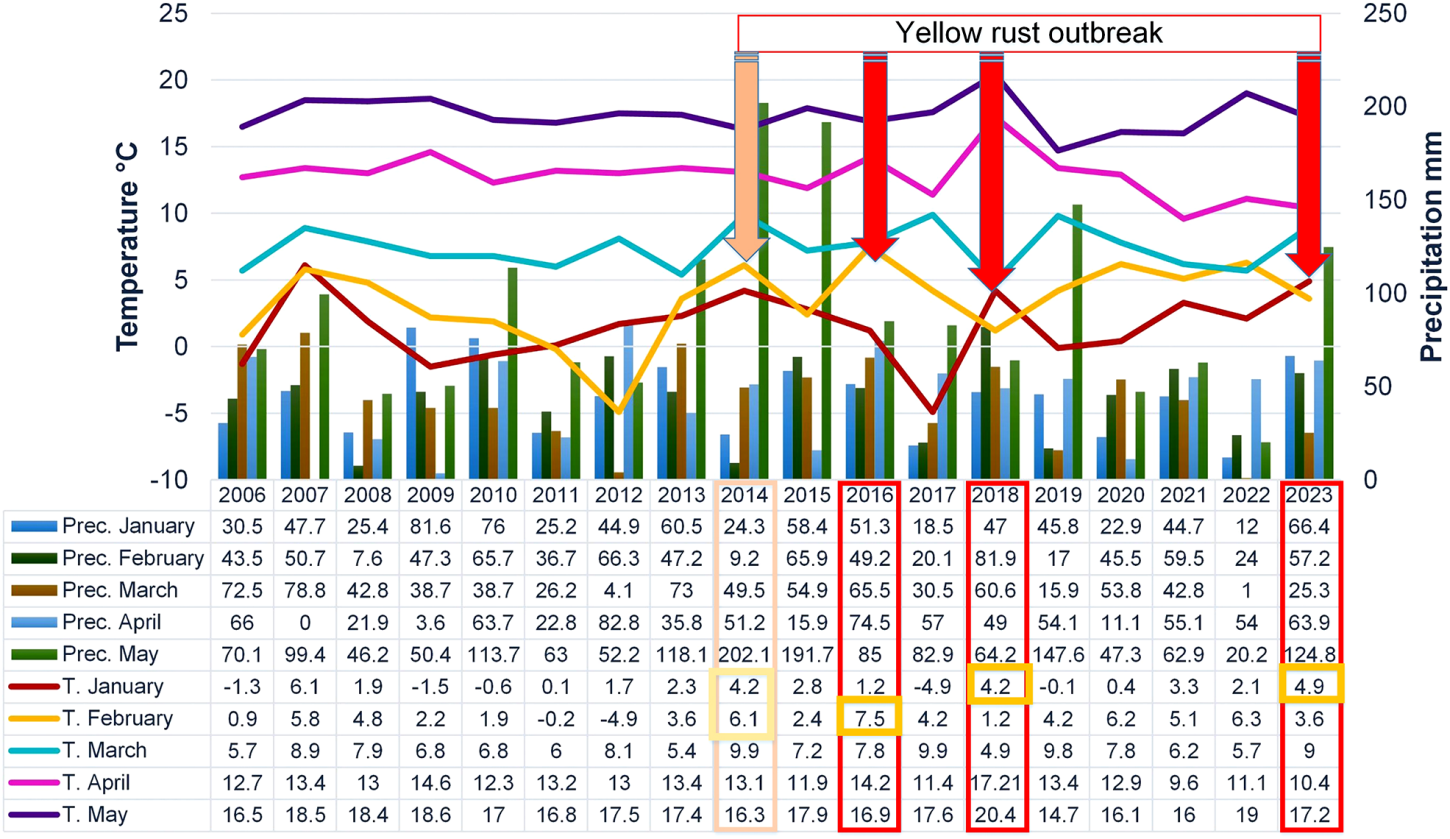
Climatic factors in the phenotyping platform at Rimski šančevi from 2006 to 2023 and yellow rust outbreaks in Serbia.

Monthly mean temperatures and precipitation in the phenotyping platform were recorded for each growing season (from January to May) using data from the Republic Hydrometeorological Service of Serbia (http://www.hidmet.gov.rs/) (**Figure 1**). The weather station was placed on the same locality were the trials were conducted. On average, from 2016 to 2023, April was the least humid month in Novi Sad, with a relative humidity of 64.5%. Since it was still conducive enough for infection and development of rust diseases (El Jarroudi et al., 2017), relative humidity was not taken into account in the regression and PCAmix analysis. To provide a broader insight into climatic trends in Serbia, we are presenting the climatic conditions at the Rimski šančevi locality from 2006 to 2023 (**Figure 1**). The data on the outbreak of yellow rust in 2014 have already been published (Jevtić et al., 2017) and were not included in this study.

The minimum, optimum, and maximum temperatures affecting different stages of the life cycle of yellow and leaf rust (Singh et al., 2002) are provided in **Table 1**.

**Table 1:** Minimum, optimum and maximum temperatures affecting life cycle of yellow and leaf rust.

	Leaf rust	Yellow rust	Leaf rust	Yellow rust	Leaf rust	Yellow rust	Leaf rust	Yellow rust
	Germination		Penetration		Growth		Sporulation	
Minimum T	2	0	10	2	2	3	10	5
Optimum T	20 12-15	9-13	20	9-13	25	12-15	25	12-15
Maximum T	30	23	30	23	35	20	35	20

### 2.4 Statistical methods

Associations between qualitative variables (year) and quantitative variables (genotype reaction to yellow and leaf rust and climatic factors) were analyzed using principal component analysis with mixed data (PCAmix) since PCA requires quantitative variables and MCA requires qualitative variables only.

The factors influencing dependent variables (the disease index of leaf and yellow rust) were analyzed using a multiple linear stepwise regression model due to multicollinearity of the data and best subsets regression. The best subsets regression was performed to identify the best-fitting regression models with predictors of choice (abiotic and biotic factors) analyzed individually and in combination with each other. Genotypes were considered as categorical variables in multiple linear regression models, while obligate pathogens and climatic factors (temperature and total rainfall) were considered as continuous ones. Obligate pathogens were included in regression modelling as independent variables since different rust species can be habitant of the same host plant. The alpha level to enter and alpha level to remove the influencing factors in the stepwise regression were set by default to 0.15 since it was reported that an alpha level of 0.05 could fail to identify important variables (Bursac et al., 2008).

Regression models were followed with the coefficient of determination (R^2^), which is the percentage of variation in the response that is explained by the model. The analysis was performed using XLSTAT in Microsoft Excel (XLSTAT Statistical and Data Analysis Solution, 2022) and Minitab 17 Statistical Software (trial version). Package ‘ggplot2’ in R software was used for the visualization of PCAmix analysis (RStudio Team, 2022).

## 3 Results

**Figure 2:**
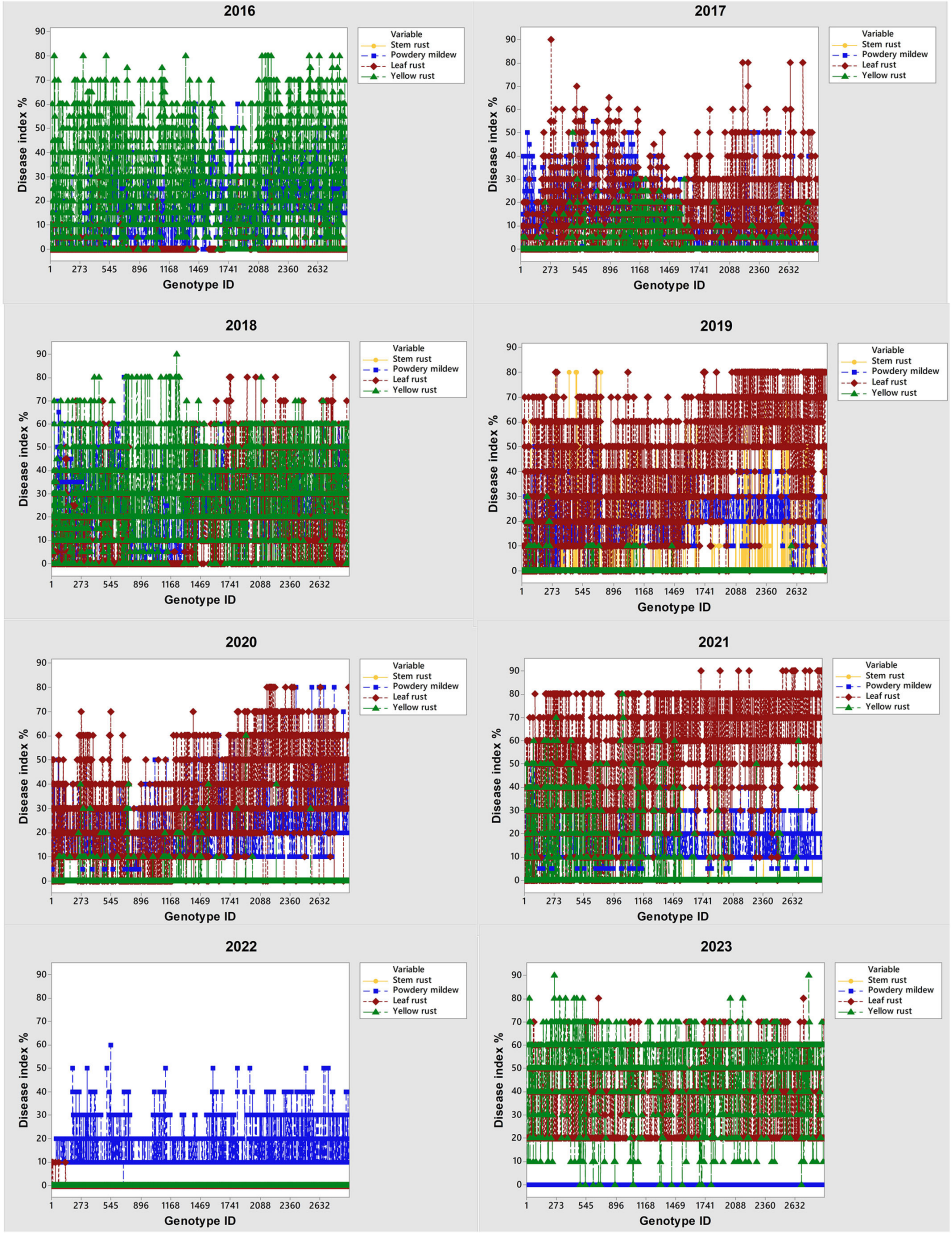
Changes in predominance of obligate pathogens (powdery mildew, leaf rust, yellow rust and stem rust) on 2715 winter wheat genotypes and wild relatives from 2016 to 2023. Phenotyping platform is located on Rimski šančevi (Vojvodina—north province of Serbia).

In the period 2016-2023, the occurrence of the obligate pathogens in 2715 genotypes was not straightforward (**Figure 2**; **Table 2**). The predominance of yellow rust over leaf rust in the phenotyping platform had been recorded in 2016, 2018, and 2023 when 72%, 78%, and 96.5% of the genotypes were infected with yellow rust with a disease index exceeding 10%, respectively. In the rest of the years, no more than 11% of genotypes were infected with yellow rust. The average disease indices of yellow rust in 2016, 2018, and 2023 were 28.3%, 30.2%, and 52%, respectively (**Table 2**). It should be pointed out that in all years with yellow rust outbreak in the phenotyping platform at Rimski šančevi temperatures in January and/or February exceeded the eighteen-year average by 2.7°C (**Figure 1**).

**Table 2:** Mean, minimum, median and maximum disease indices of obligate pathogens (powdery mildew, leaf rust, yellow rust and stem rust) in the phenotyping platform in Rimski šančevi from 2016 to 2023.

Obligate pathogen	Year	Mean DI	Minimum DI	Median DI	Maximum DI
Stem rust	2016	0.0	0.0	0.0	0.0
	2017	0.0	0.0	0.0	0.0
	2018	0.0	0.0	0.0	0.0
	2019	8.6	0.0	0.0	80
	2020	0.0	0.0	0.0	0.1
	2021	0.1	0.0	0.0	60
	2022	0.00004	0.0	0.0	0.1
	2023	0.0	0.0	0.0	0.0
Powdery mildew	2016	13.8	0.0	10	60
	2017	10.6	0.0	10	60
	2018	22.2	0.0	20	80
	2019	17.7	0.0	20	60
	2020	20.7	5.0	20	80
	2021	13.8	5.0	10	60
	2022	14.5	0.0	10	60
	2023	0.0	0.0	0.0	0.0
Leaf rust	2016	0.5	0.0	0.0	45
	2017	13.5	0.0	10	90
	2018	14.5	0.0	0.1	80
	2019	40.9	0.0	40	80
	2020	31.9	0.0	30	80
	2021	53.1	0.0	60	90
	2022	0.5	0.0	0.0	10
	2023	34.5	20	40	80
Yellow rust	2016	28.3	0.0	25	80
	2017	2.6	0.0	0.0	50
	2018	30.2	0.0	30	90
	2019	0.2	0.0	0.0	30
	2020	0.5	0.0	0.0	60
	2021	4.1	0.0	0.0	80
	2022	0.0003	0.0	0.0	0.1
	2023	51.9	0.1	50	90

The average disease indices of powdery mildew ranged from a trace level in 2023 to 22.2% in 2018. *P. triticina* was the predominant rust species in the years that were not conducive to yellow rust occurrence, except in 2022 when powdery mildew was the prevalent pathogen. In the years not conducive to yellow rust occurrence, the average disease DI of leaf rust was 13.5% in 2017, 40.8% in 2019, 31.85% in 2020, 53.1% in 2021 and 0.5% in 2022 (**Table 2**). The predominance of powdery mildew over rust diseases in 2022 can be attributed to the colder weather and significantly lower spring precipitation compared to the seasonal average (**Figure 1**). It is known that powdery mildew and leaf rust require different conditions for successful development. Powdery mildew typically emerges as the first leaf disease in spring, thriving in temperatures between 10 and 21°C. In the agro-ecological conditions of Serbia, it can even be observed as early as February. In contrast, optimal conditions for leaf rust initiation are usually met in the first half of April (Jevtić et al., 2020). Additionally, powdery mildew is unique compared to other fungi since its spores do not require dew or rain for germination (Salgado and Paul, 2016). Since the spring of 2022 (March-May) began with dry weather conditions, and recorded precipitation was minimal, with just 1 mm measured in March at the experimental site (**Figure 1**), it gave advantage to powdery mildew development over rust diseases. Although the weather was sunnier and warmer in May than usual, it remained dry. Rainfall was mostly recorded during the final 10 days of the month, with an average total precipitation of 20.2 mm, which was 70 mm below the eighteen-year average of 91.2 mm (**Figure 1**). All these factors resulted in the predominance of powdery mildew in 2022.

Stem rust outbreaks in the phenotyping platform occurred only in 2019 when 32% of genotypes were infected with DI ranging from a trace level to 80%. Disease indices of stem rust above 10% were recorded in 18% of genotypes in 2019. In 2021, stem rust occurred only in 0.9% of genotypes with DI ranging from a trace level to 60%. Jevtić et al. (2020) reported that the delayed development of winter wheat genotypes, caused by extreme fluctuations in total rainfall during the winter period, can significantly increase their susceptibility to stem rust.

The range of disease indices of each obligate pathogen in the phenotyping platform indicates great diversity in susceptibility responses of genotypes to obligate pathogens. As a consequence, a more detailed analysis of factors affecting shifts in the predominance of leaf and yellow rust, increasing levels of coexistence of leaf and yellow rust, and yellow rust resistance breakdown are discussed in more detail further.

### 3.1 Shifts in predominance of leaf and yellow rust

**Figure 3:**
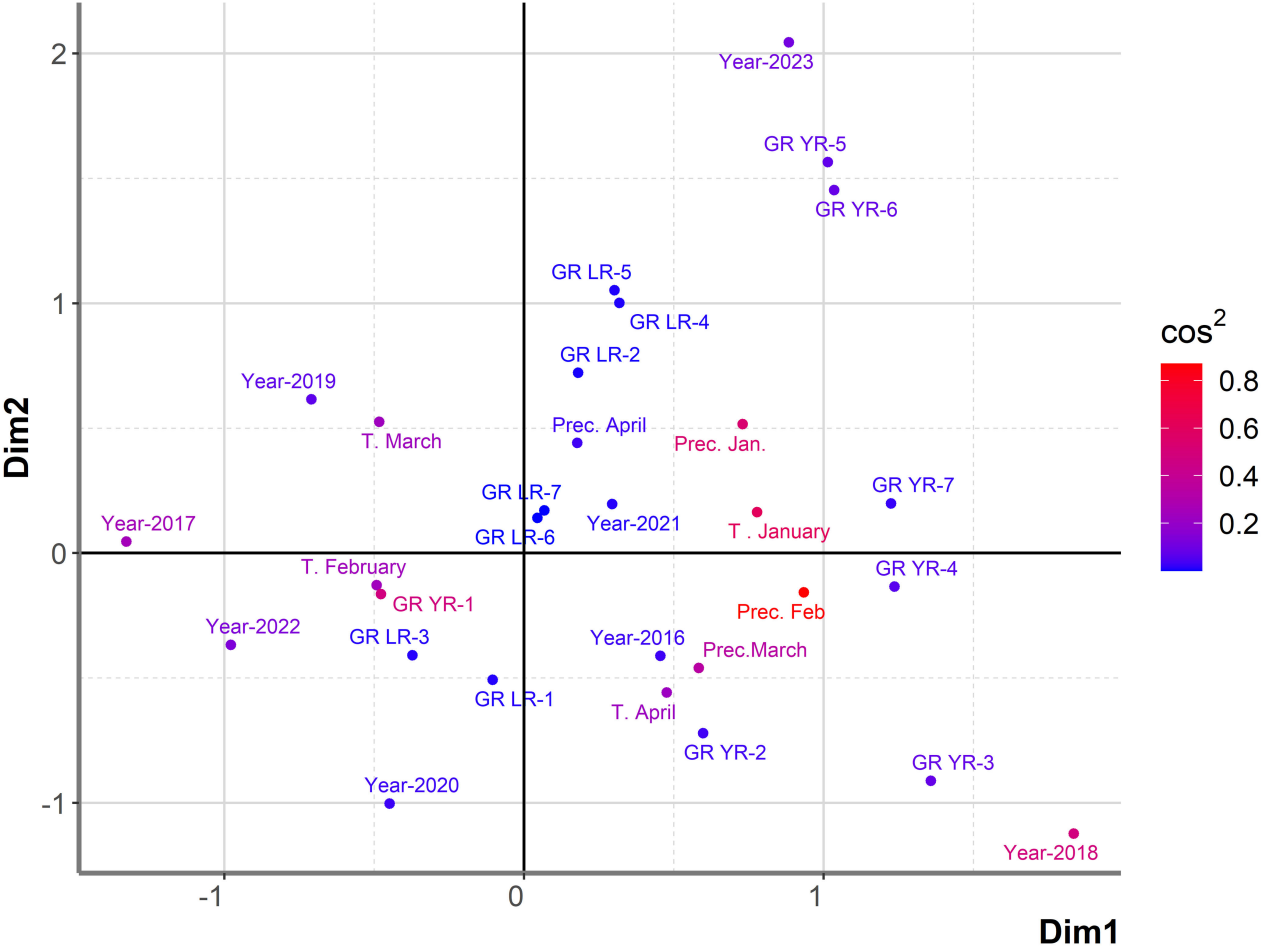
Graphical representation of PCAmix analysis on subset of 764 genotypes showing susceptibility to rust diseases in the period from 2016 to 2023. Factors included were: years (from 2016 to 2023), temperatures and precipitations from January to April from 2016 to 2023, reaction of 764 genotypes to leaf rust (GR LR) and reaction of 764 genotypes to yellow rust (GR YR).

To exclude the effect of powdery mildew on the relationship between leaf, yellow rust, and host plants, influencing factors on shifts in the predominance of leaf and yellow rust were analyzed using a set of 764 genotypes where the disease index of powdery mildew did not exceed 20% (**Supplementary Table 1**). The general association between years, genotype’s reactions to leaf and yellow rust, and climatic factors from January to April in an eight-year period was examined using PCAmix analysis (**Figure 3**). Since assessments of rust diseases were made in May/June when temperatures exceeded 15°C and slowed down yellow rust development, climatic conditions in May are excluded from PCAmix analysis.

In the subset of 764 genotypes, DI of yellow rust exceeding 41% (GR-5, GR-6) was highly associated only with 2023, indicating a higher pressure of yellow rust on wheat genotypes in 2023 than in previous years. In 2023, 81% of 764 genotypes were infected with yellow rust with DI exceeding 41%. In addition, leaf rust with moderate level of infection (from 31% to 50%) was associated with both 2023 and 2021, indicating a higher level of coexistence of leaf and yellow rust in 2023. Contrary to 2023, there were 8.6% and 23% of genotypes infected with yellow rust with DI exceeding 41% in 2016 and 2018, respectively. It resulted in the position of 2016 and 2018 on the opposite side of the factor map from 2023. A low level of yellow rust infection occurred in 2020 and 2022, with DI not exceeding 10% (2022) and 20% (2020). In these years, DI of leaf rust in the majority of genotypes was also at a low (GR-1) to moderate level of infection (GR-3). In the rest of the years (2017, 2019, and 2021), leaf rust was the predominant pathogen with disease indices exceeding 51%, especially in 2021.

Temperature in March was indicated as the most influencing factor on leaf rust infection in 2017 and 2019, while precipitation in April was the most associated with leaf rust infection in 2021. Temperature in January and precipitations in January were associated with the highest level of infection with yellow rust (GR-7) (**Figure 3**). The first two dimensions contributed 28.8% to the overall variability. The contribution of the first and second dimensions was 18.1% and 10.7%, respectively. Since PCAmix is used as a tool in exploratory data analysis, further analysis was performed with regression modeling.

Regression modelling of the most influencing factors on yellow rust infection in 764 genotypes confirmed that T in January (P<0.001), T in April (P<0.001), precipitation in January (P<0.001), precipitation in February (P<0.001), precipitation in March (P<0.001), leaf rust infection and genotype (P<0.001) significantly affected yellow rust occurrence giving the regression model with R^2^ of 65.5% (**Table 3**). The same as in PCAmix analysis, regression modelling indicated significant effect of T in February (P<0.001), T in March (P=0.003) and total rainfall in April (P<0.001) on leaf rust occurrence in 764 genotypes. The genotype (P<0.001) and yellow rust infection (P<0.001) also affected leaf rust infection as shown by regression modelling but R^2^ did not exceed 31% so there are additional factors that affected differences in leaf rust infection in phenotyping platform (**Table 3**).

**Table 3:** Regression modelling on the most influencing factors on yellow and leaf rust infection in 764 genotypes from 2016 to 2023.

Source	DF	Adj SS	ADj MS	F-Value	P-Value
Yellow rust					
Regression	769	1708410	2222	11.13	<0.001
T in January	1	53456	53456	267.75	<0.001
Prec. in January	1	301038	301038	1507.85	<0.001
Prec. in February	1	174997	174997	876.53	<0.001
Prec. in March	1	74176	74176	371.54	<0.001
T in April	1	16122	16122	80.75	<0.001
Leaf rust	1	143925	143925	720.90	<0.001
Genotype	763	281732	369	1.85	<0.001
Error	4499	898212	200		
Total	5268	2606622			
Leaf rust					
Regression	767	911665	1188.6	2.61	<0.001
Prec. in April	1	6586	6586.0	14.48	<0.001
T in March	1	3915	3915.1	8.61	0,003
T in February	1	27980	27979.9	61.52	<0.001
Yellow rust	1	40065	40065.2	88.09	<0.001
Genotype	763	789683	1035.0	2.28	<0.001
Error	4501	2047045	454.8		
Total	5268	2958711			

In our study, the response of 764 susceptible genotypes to leaf and yellow rust over an eight-year period exhibited considerable dissimilarities, not only in terms of one pathogen’s prevalence over the other but also in their coexistence. Although regression modeling indicated that fluctuations in January and April temperatures, as well as precipitation in January, February, and March, affected yellow rust occurrence on the 764 genotypes during the eight-year period, none of these factors were specifically associated with the year 2023 when the coexistence of both pathogens occurred. Consequently, we can assume that the reaction of the 764 genotypes to climatic conditions in 2023 was rather diverse, resulting in variability in their response to leaf and yellow rust infections. This also suggests that a more specific dataset should be analyzed to evaluate which climatic factors were most favorable in promoting the coexistence of both pathogens in the same year.

**Figure 4:**
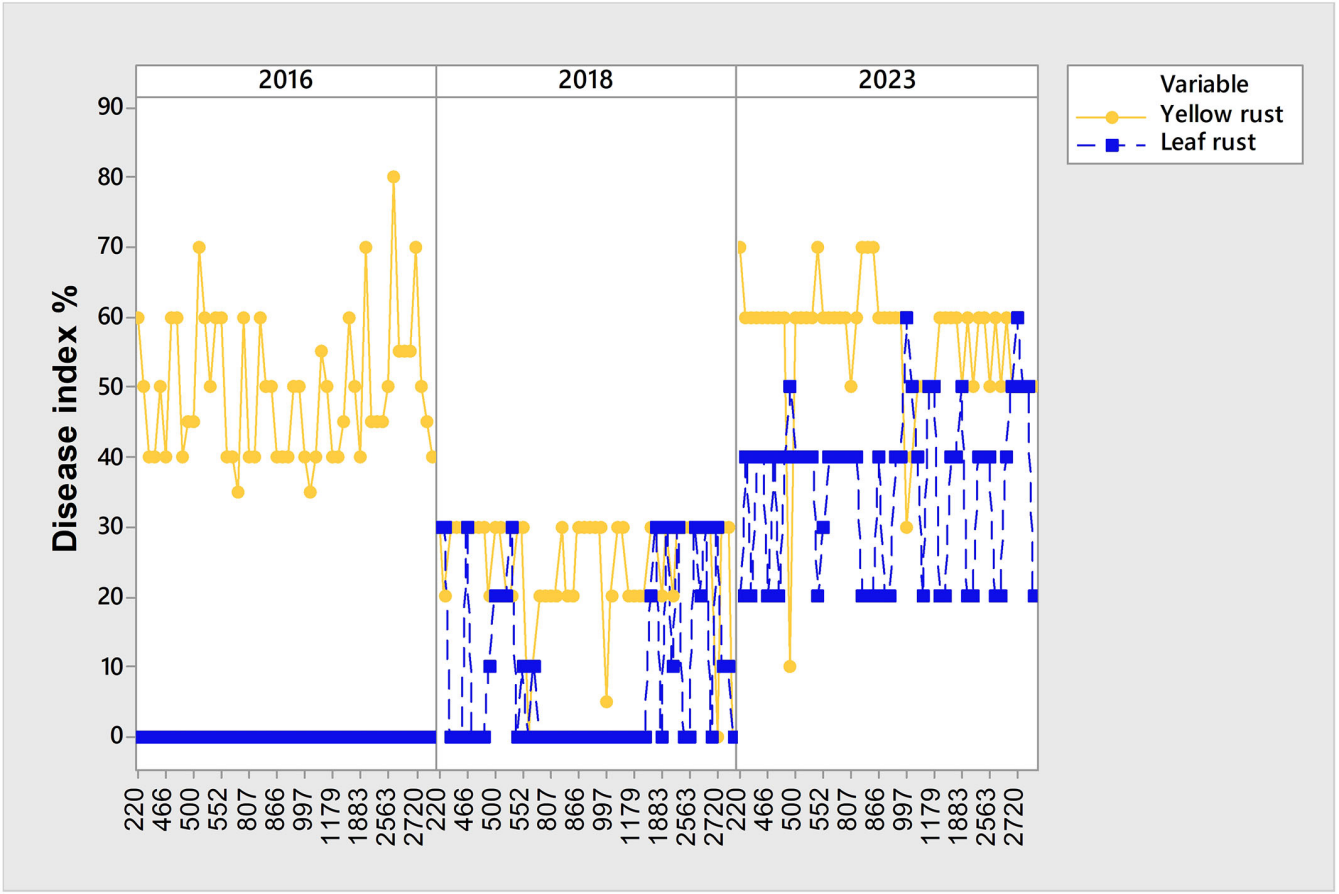
Set of 54 susceptible genotypes to yellow rust showing different levels of susceptibility in 2016, 2018 and 2023 that were conducive for yellow rust occurrence.

It should be pointed out that although 2016, 2018, and 2023 were favorable years for yellow rust infection, there was a set of genotypes susceptible to yellow rust that showed different levels of susceptibility reactions over the three-year period, despite it being known that yellow rust is a more aggressive pathogen. All of them were susceptible to yellow rust in 2016 and 2023, but in 2018 showed moderate levels of susceptibility with DI below 30% (**Figure 4**). General liner modeling confirmed that yellow rust infection was significantly affected not only by year and competing leaf rust but also by genotype itself (**Table 4**).

**Table 4:** General linear modelling on the most influencing factors on yellow rust infection in 54 genotypes in 2016, 2018 and 2023.

Source	DF	Adj SS	ADj MS	F-Value	P-Value
Leaf rust	1	466.0	466.0	7.37	0.008
Year	2	26930.7	13465.3	212.93	P<0.001
Genotype	53	7004.0	132.2	2.09	0.001
Error	104	6576.9	63.2		
Total	160	44871.1			

Since PCAmix indicated the shifts in the predominance of leaf and yellow rust on 764 genotypes over an eight-year period, as well as increased levels of yellow rust infection together with the coexistence of leaf and yellow rust in 2023, further analysis on factors influencing yellow rust infection was conducted in two sets of genotypes showing: 1) coexistence between yellow and leaf rust in 2023 but not in 2016 and 2018; and 2) susceptibility reactions to yellow rust only in 2023.

### 3.2 Increased level of coexistence of leaf and yellow rust

**Figure 5:**
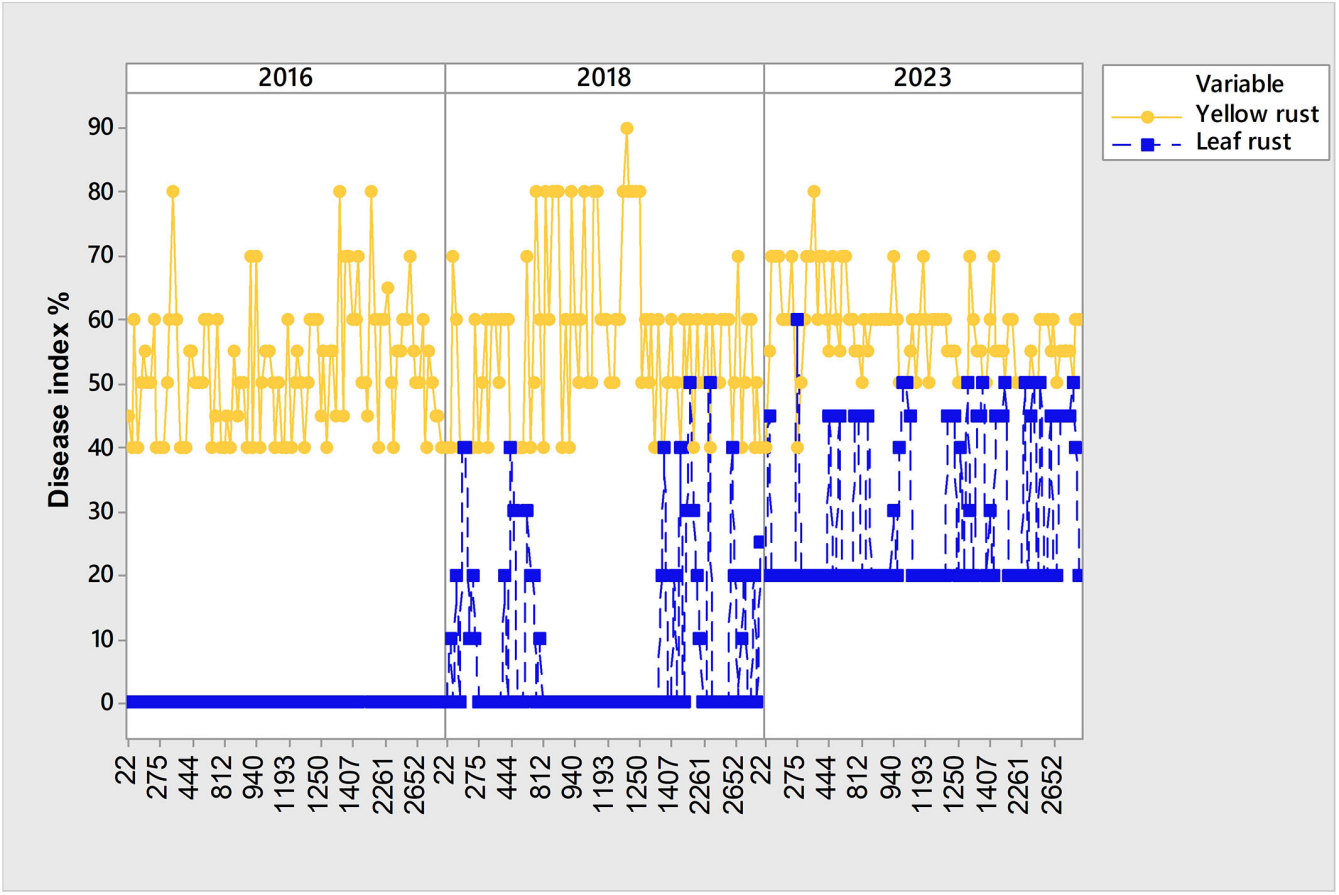
Set of 99 susceptible genotypes to yellow rust in 2016, 2018 and 2023 with higher level of coexistence of leaf and yellow rust in 2023.

Although yellow rust is known to be more aggressive than leaf rust, and despite favorable climatic conditions in 2016, 2018, and 2023 for yellow rust infection, the coexistence of yellow and leaf rust increased in 2023 when compared with 2018 and 2016 (**Figure 2**; **Figure 5**). Consequently, the subset of 99 genotypes (**Supplementary Table 1**) with a DI of yellow rust exceeding 41% in 2016, 2018, and 2023, and showing coexistence between leaf and yellow rust in 2023 (**Figure 5**), was analyzed using PCAmix and regression modeling to find out which climatic factors were most associated with leaf rust infection.

**Figure 6:**
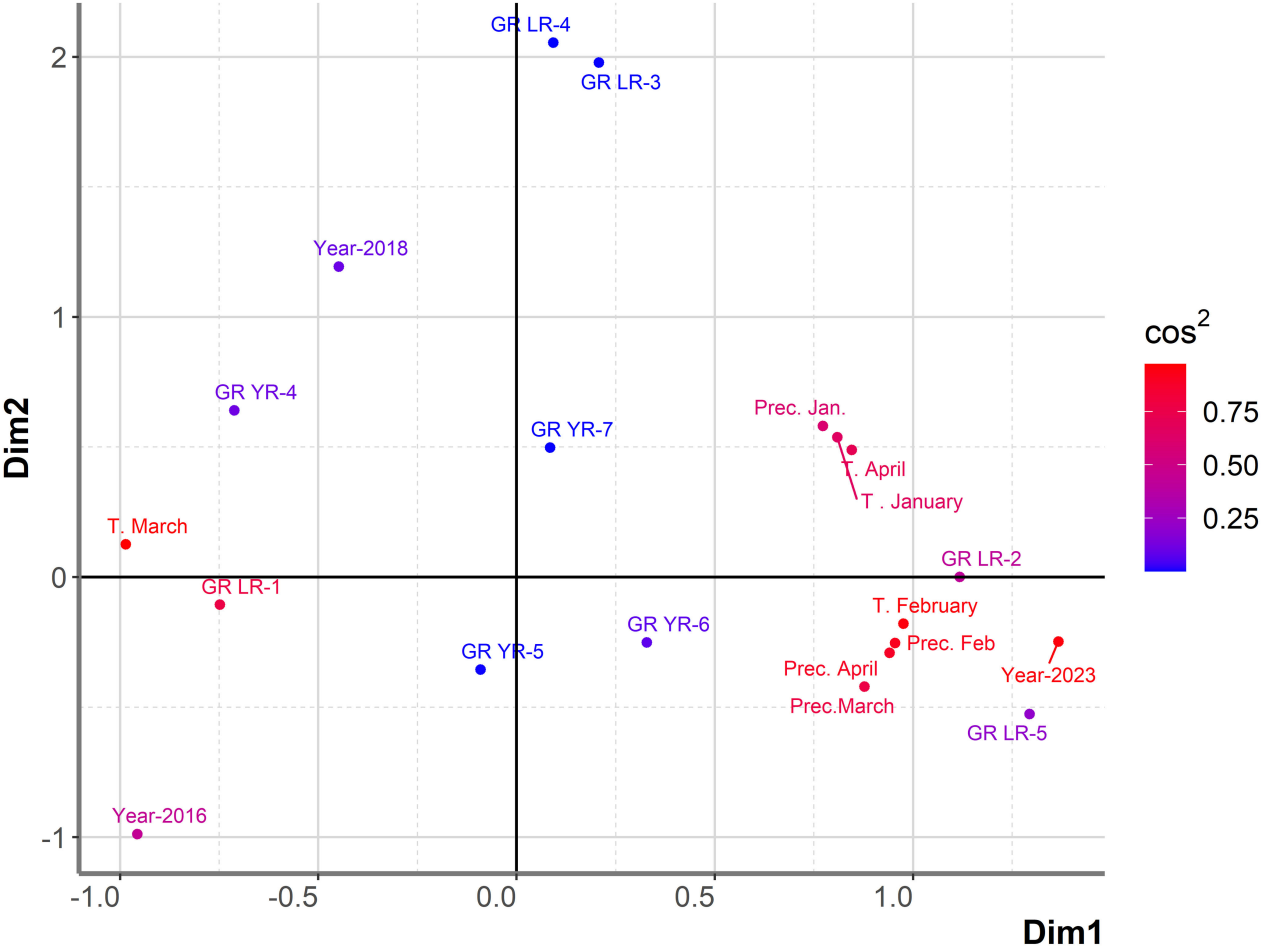
Graphical representation of PCAmix analysis on subset of genotypes showing coexistence between leaf and yellow rust in 2023. Factors included were: years (2016, 2018 and 2023), temperatures and precipitations from January to April in 2016, 2018 and 2023, reaction of 99 genotypes to leaf rust (GR LR) and reaction of 99 genotypes to yellow rust (GR YR).

PCAmix analysis indicated that T in February, as well as precipitation in February, precipitation in March, and precipitation in April, significantly affected both leaf and yellow rust infection in the subset of 99 genotypes showing coexistence of leaf and yellow rust in 2023 (**Figure 6**). The first two dimensions contributed 60.7% to the overall variability. The contribution of the first and second dimensions was 49.4% and 11.3%, respectively. In the larger set of genotypes (764) in eight-year period, T in February and precipitation in April significantly affected leaf rust infection, while precipitation in February and precipitation in March were mostly associated with moderate levels of yellow rust infection.

Since PCAmix was used for reducing the dimensionality of a dataset, two types of regression modeling were carried out to estimate relationships between dependent variable (leaf rust infection) and independent variables (genotype, climatic factors, and competing yellow rust). For explaining leaf rust infection in the years conducive to yellow rust infection, the best subsets regression indicated that T in February, total rainfall in March, and DI of yellow rust formed the model with the smallest Mallows’ Cp (4), meaning that this model is relatively unbiased in estimating the true regression coefficients and predicting leaf rust infection (**Table 5**). Additionally, the best subsets regression showed that using only biotic or abiotic factors for regression modeling and prediction of leaf rust infection would give lower R^2^, R^2^_pred_, and a much higher Mallows’ Cp than using them as combined predictor variables.

**Table 5:** Best subsets regression for predicting leaf rust infection in 99 varieties in the years conducive for yellow rust occurrence (2016, 2018 and 2018).

Number of predictors	R^2^	R^2^_pred_	Mallows’ Cp	T in February (°C)	Precipitation in March(mm)	Yellow rust DI index (%)
1	53.6	52.9	65.2	X		
1	41.8	40.9	154.8		X	
2	57.4	56.5	38.1	X	X	
2	57.1	56.2	40.3	X		X
3	62.2	61.1	4.0	X	X	X

Considering that the minimal temperature required for leaf rust penetration and sporulation is 10°C, typically achieved in April under Serbia’s agro-ecological conditions, and that the latent period of leaf rust can be extended at temperatures between 5-10°C, it is plausible to suggest that the February temperature (3.6°C) and the below-average total rainfall in March (25 mm) created conditions more favorable for yellow rust infection and also delayed the development of leaf rust. Since duration of wheat phenological phases are also highly associated with earliness of genotypes and climatic conditions it can be inferred that these factors created an environment where leaf rust could thrive on the remaining green leaf tissue of susceptible genotypes only when the development of yellow rust was slowed down.

Stepwise regression confirmed the same set of predictors as significantly influencing leaf rust infection (P<0.001), together with the effect of genotype as a categorical predictor, giving the model with R^2^ of 77% (**Table 6**).

**Table 6:** Regression modelling on the most influencing factors on leaf rust infection in 99 genotypes in the years conducive for yellow rust occurrence (2016, 2018 and 2018).

Source	DF	Adj SS	ADj MS	F-Value	P-Value
Regression	101	58823	582.4	6.24	P<0,001
T in February	1	13060	13060.4	139.95	P<0,001
Prec. in March	1	3498	3498.4	37.49	P<0,001
Yellow rust	1	2957	2956.8	31.68	P<0,001
Genotype	98	11286	115.2	1.23	0.111
Error	189	17638	93.3		
Total	290	76461			

Since DI of yellow rust in the subset of 99 genotypes did not change significantly in 2016, 2018 and 2023, we did not conduct analysis to find out if there are some additional climatic factors affecting yellow rust occurrence besides those obtained by PCAmix analysis.

### 3.3 Yellow rust resistance breakdown in 2023

**Figure 7:**
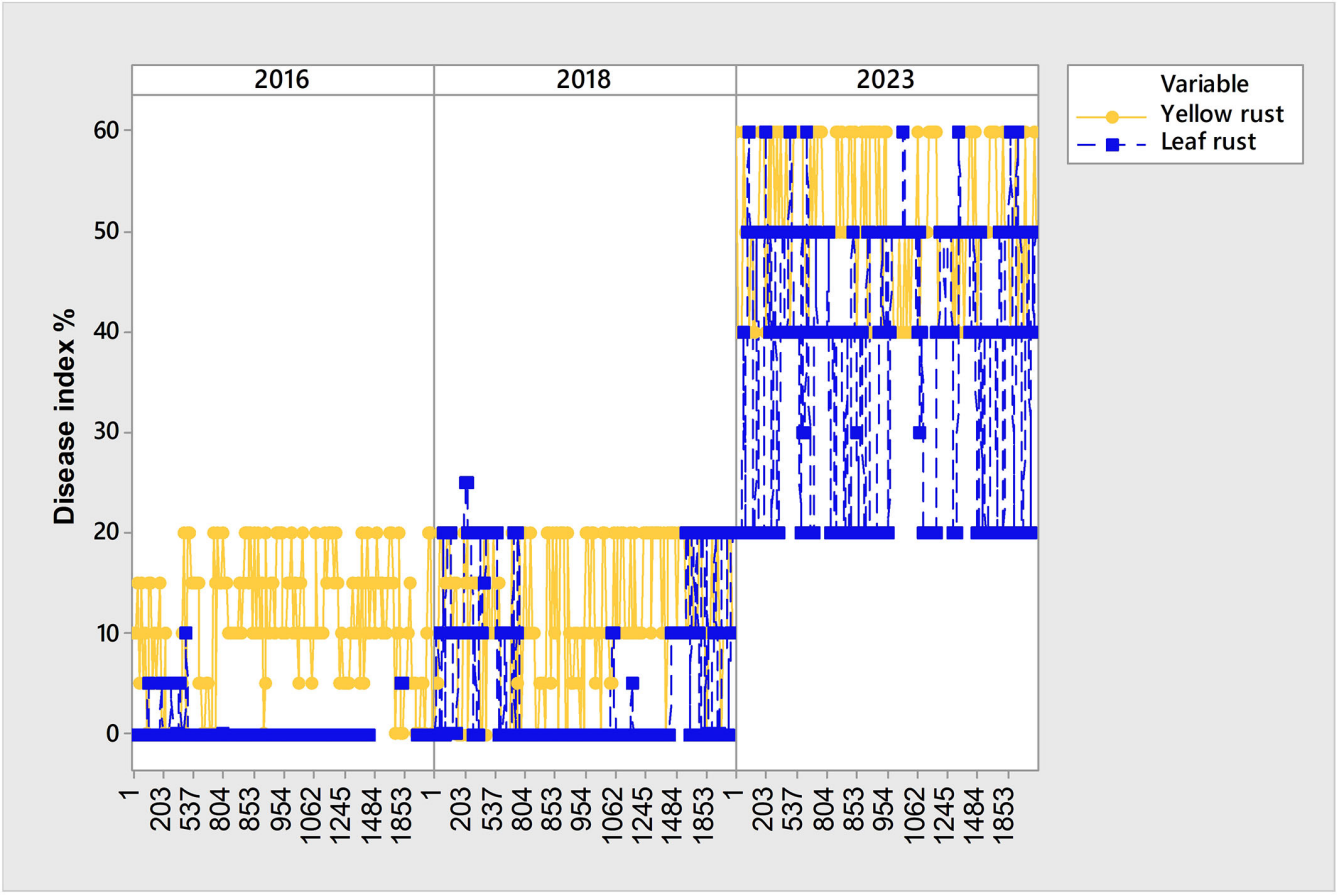
Set of 180 genotypes showing leaf and yellow rust resistance breakdown in 2023.

In the phenotyping platform, there were 6.6% of genotypes (180 out of 2715) highly to moderately resistant to yellow rust in 2016 and 2018, but with yellow rust resistance breakdown in 2023, with yellow rust DI ranging from 40% to 60% (**Figure 7**; **Supplementary Table 1**). The same set of genotypes was resistant or moderately resistant to leaf rust in 2016 and 2018 as well.

Yellow rust resistance breakdown in 2023 was recorded in wild relatives as well as in winter wheat genotypes having all-stage (AS) race-specific genes (Lr1, Lr3, Lr10, Lr14a, Lr2a, Lr24, Yr2, Yr4, Yr6, Yr7, Yr9) and adult plant resistance genes (Lr13, Lr20, Yr14, Lr34). Wild relatives that showed susceptibility reaction to yellow rust in 2023 were: *Triticum paleocolchicum* Men.; *Triticum macha* var. *subletschchumicum*; *Triticum macha* var. *paleo-imereticum*; *Triticum dicoccoides* var. *aaronsohni* Flaksb; *T. macha* var. *submegrelicum* Dekapr. & Menabde; *Triticum dicoccum* Schrank ex Schübl. var. *atratum; Triticum dicoccum* var. *farum* Ja; *Triricum dicoccum* var. *rufum* (Schübl.); *Triticum durum* Desf. var. *affine* (Korn.); *Triticum polonicum* var. *vestitium*; *Triticum polonicum* L. var. *gracile* Flaksb; *Triticum polonicum* var. *villosum* Desv.

**Figure 8:**
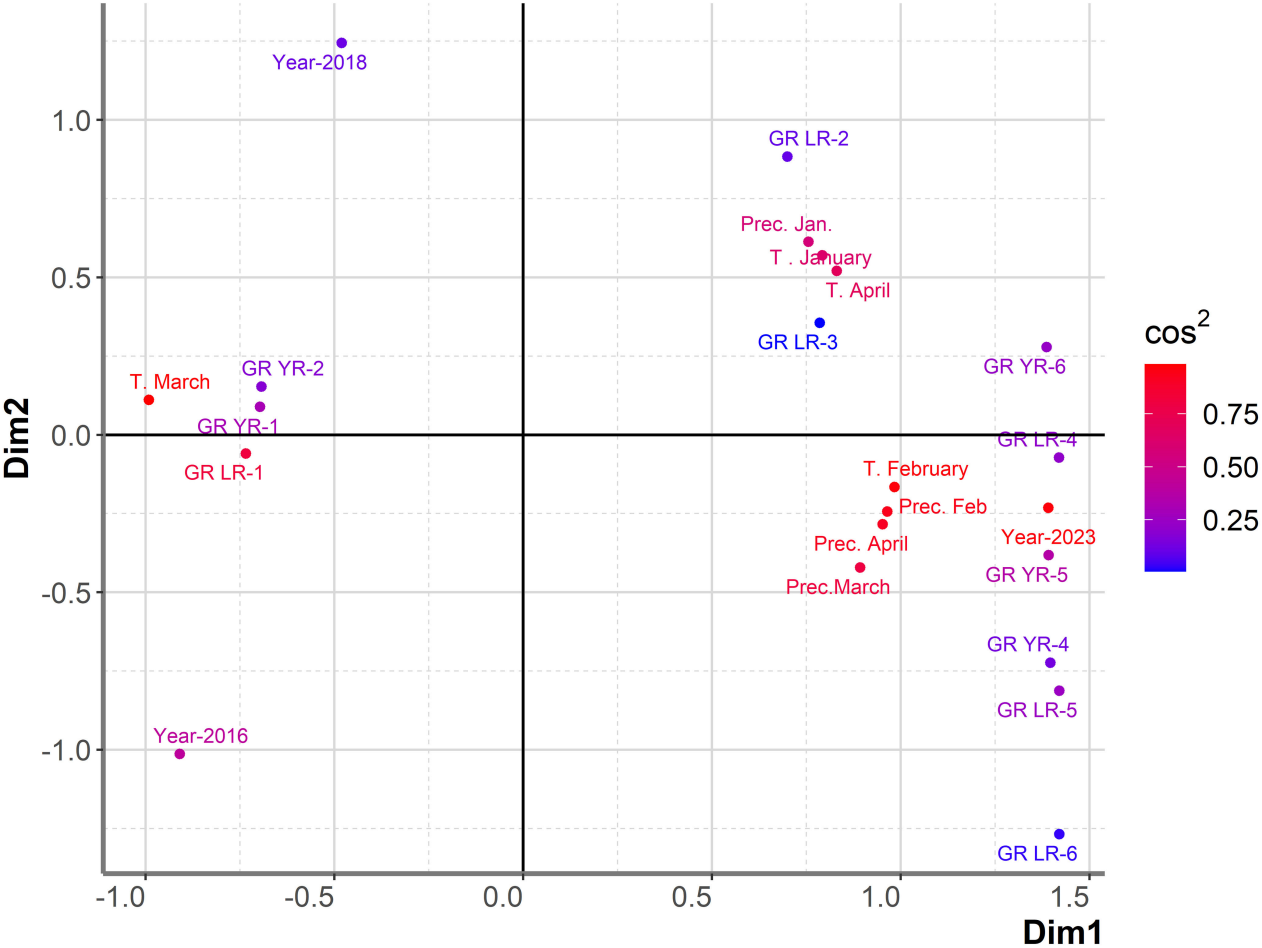
Graphical representation of PCAmix analysis on subset of genotypes showing resistance breakdown to leaf and yellow rust in 2023. Factors included were: years (2016, 2018 and 2023), temperatures and precipitations from January to April in 2016, 2018 and 2023, reaction of 180 genotypes to leaf rust (GR LR) and reaction of 180 genotypes to yellow rust (GR YR).

PCAmix indicated that T in January, precipitation in January, and T in April were associated with the highest level of infection with yellow rust (GR-6) (**Figure 8**). The first two dimensions contributed 58.4% to the overall variability. The contribution of the first and second dimensions was 48.8% and 9.6%, respectively. The same combination of climatic factors, with the exception of T in April, was associated with high levels of yellow rust infection in the set of 764 genotypes analyzed over an eight-year period. In that dataset T in April was more associated with a moderate level of yellow rust. However, T in February and precipitation in April, which highly affected leaf rust infection over the eight-year period, appeared to influence both leaf and yellow rust infection in the subset of genotypes showing yellow and leaf rust breakdown in 2023 (**Figure 8**). Precipitation in February and precipitation in March, which were associated with moderate levels of yellow rust infection in the eight-year period, were shown to affect leaf and yellow rust infection in 2023 with DI exceeding 31%. These results indicated that resistance breakdown to yellow rust was not associated with any specific climatic factors that were not already shown to be favorable for yellow rust infection of larger dataset comprising of 764 genotypes. Consequently, a more detailed study on changes in the race structure of yellow rust in the phenotyping platform and the individual response of each yellow rust resistance gene should be investigated in the future to explain the causes of yellow rust resistance breakdown in 2023.

## 4 Discussion

The results of this study indicated that the relationship between leaf and yellow rust is not straightforward and is strongly influenced not only by prevalent races and climatic factors affecting life cycle of pathogens, but also by differences in susceptibility reactions of wheat genotypes to agro-ecological conditions. In this study, we showed that the response of genotypes lacking resistance genes to yellow rust is not uniform in the years favorable for yellow rust infection, and we also observed the tendency of coexistence of leaf rust and yellow rust in the same host plant, although yellow rust is considered more aggressive. The effectiveness of resistance genes to rust diseases in Serbia is missing in reports related to European countries, making this study the first of its kind. Our study finally pointed out that resistance responses to rust diseases should not be investigated solely through the interaction between resistance genes and pathogen virulence capabilities but also through plants’ response to combined abiotic and biotic stressors, which should be treated as a unique condition.

### 4.1 Shifts in predominance of leaf and yellow rust

The effect of climatic conditions on life cycle of the pathogens and susceptibility of host plants were always considered as primary precondition for pathogens outbreak. It is known that rust diseases have different requirements for initiation of infection and disease development, and that yellow rust is favored by elevated temperatures during the winter period (Hovmøller et al., 2016).

From 2016 to 2023, Serbia experienced extreme fluctuations in climatic elements, with temperatures in January and February varying by almost 10°C (ranging from -5 to +5°C in January) and 7°C (ranging from 1.2 to 7.5°C in February). In addition, there was a difference in total precipitation ranging from 1 to 65.5 mm in March and 11.1 to 74.5 mm in April. The results of our study indicated that winter temperatures (more specifically T in January) significantly affected yellow rust occurrence over the eight-year period. Although T in February was more associated with differences in leaf rust infection among years, in 2016, T in February exceeded the eighteen-year average by 4°C, setting the precondition for yellow rust predominance over leaf rust. In 2016, leaf rust infected only 4.8% of genotypes with DI above the trace level. PCAmix and regression modeling also indicated that precipitation in February and precipitation in March are associated with moderate levels of yellow rust infection. In the years with higher levels of yellow rust infection, total precipitation in February and/or March exceeded the eighteen-year average. This study’s regression modeling on the most influencing factors on leaf rust infection gave an R^2^ that did not exceed 31%, so a more specified dataset should be defined to explain differences in the effect of climatic factors on leaf rust infection. In the years with lower DI of leaf and yellow rust, extremely low T in January (-5°C) (2017) and lack of total rainfall in March (1 mm) (2022) resulted in the predominance of leaf rust over yellow rust in but with a lower DI than in the rest of the years. Powdery mildew predominated over all rust species in 2022 due to very dry conditions.

Jevtić et al. (2020) highlighted the significance of genotype reactions to climatic elements during specific phenological stages as crucial factors affecting interactions among obligate pathogens and the dominance of one pathogen over another. The study also emphasized the need for increased attention to understanding how winter wheat genotypes can escape severe yellow rust infections during years initially conducive to pathogen development. Notably, the research revealed that, in the agro-ecological conditions of Serbia, elevated temperatures in February could lead to a higher prevalence of yellow rust in a larger number of susceptible winter wheat genotypes, compared to years where such high winter temperatures are observed only in January. The results of our study supported those of Jevtić et al. (2020) showing variability in the susceptibility reaction to yellow rust of a single genotype in years conducive to yellow rust occurrence. Significant effect of genotype itself was shown in the set of genotypes having high level of susceptibility to yellow rust in 2016 and 2023, but not in 2018 that was also conducive for yellow rust infection. In addition, the reaction of 764 susceptible genotypes to leaf and yellow rust over an eight-year period was quite different, not only with respect to the predominance of one pathogen over the other but also regarding their coexistence.

### 4.2 Increased level of coexistence of leaf and yellow rust

Until now, many efforts have been made in determining damage thresholds and developing mathematical as well as threshold-based weather model for forecasting wheat disease incidence and yield losses (Junk et al., 2016; El Jarroudi et al., 2017; Jevtić et al., 2017; El Jarroudi et al., 2020; Rodríguez-Moreno et al., 2020). However, prevalence of pathogens can hardly be predicted if studies are focused only on analysis of influence of individual abiotic and/or biotic factors (White et al., 2011; Juroszek and von Tiedemann, 2013).

It is known that abiotic stressors could affect plant-pest interactions (Pandey et al., 2017). High- and low-temperature, salinity and drought are reported to affect plant physiology and defense responses (Pandey et al., 2017). It was reported that stress combinations can have negative as well as positive effects on plants since regulatory network for plant responses to abiotic and biotic stresses consists of many components that may function antagonistically, or some responses can be prioritized over others (Glazebrook, 2005; Yasuda et al., 2008; Kissoudis et al., 2014). Consequently, plant responses to combined stressors cannot be predicted if only effect of individual stresses on plant response is investigated (Mittler, 2006; Rasmussen et al., 2013; Kissoudis et al., 2014; Suzuki et al., 2014; Pandey et al., 2017). In this study, a higher level of yellow rust infection has been recorded in 2016, 2018, and 2023, but the susceptibility reactions of wheat genotypes were not uniform in years conducive to yellow rust occurrence. In the subset of 99 genotypes with a DI of yellow rust exceeding 41% in 2016, 2018 and 2023, 72% of genotypes showed coexistence between leaf and yellow rust in 2023. Contrary to that, in 2018 in the same subset of genotypes, two pathogens coexisted in 11.3% genotypes. In 2016, yellow rust predominated over leaf rust with no records of coexistence. Regression modeling indicated that the combined effect of T in February, precipitation in March, and yellow rust infection significantly affected leaf rust infection in the subset of 99 genotypes. It could be suspected that the February temperature (4.2°C), exceeding the eighteen-year average, and the total rainfall in March (25 mm), which was below the eighteen-year average, were more conducive to yellow rust infection and postponed leaf rust development, creating favorable conditions for leaf rust to colonize the remaining green leaf tissue of susceptible genotypes when the development of yellow rust was slowed down. Interestingly, 38% of the genotypes exhibiting leaf-yellow rust coexistence in 2023 were early wheat genotypes with narrow leaf blades highly susceptible to leaf rust.

It is reported that plant responses to obligate pathogens could be either independent of resistance genes and determined by temperature changes. (Bryant et al., 2014). It has also been found that plant responses to obligate pathogens could be influenced by calcium-dependent phospholipid-binding proteins responsive to both pathogen infection and temperature changes (Zou et al., 2018). However, there are still open questions regarding plant responses to the combined effects of climatic factors influencing the life cycle of pathogens, the duration of development stages of wheat genotypes, and hormonal cross-talk in plant stress responses. Considering that the regression coefficient describes the size and direction of the relationship between a predictor and the response variable, it appears from our study that further investigations should be conducted to identify predictor variables that reflect the response of individual genotypes to combined abiotic and biotic stressors. This will help in developing models for rust occurrence that can be applied to a large number of genotypes with a high level of certainty.

### 4.3 Yellow rust resistance breakdown in 2023

The breeding for resistance to rust pathogens usually takes into account race-specific and race nonspecific resistance. Race-specific resistance is conferred by a major dominant gene, expressed at all growth stages, and is not durable. The race nonspecific resistance is primarily quantitative and usually effective in later growth stages. The race nonspecific resistance is known as adult plant resistance (APR) or slow-rusting resistance (Kumar et al., 2022). In this study, 6.6% of genotypes that were resistant or moderately resistant to yellow and leaf rust in the years conducive to yellow rust occurrence (2016 and 2018) exhibited resistance breakdown in 2023. Among them, there were genotypes carrying resistance genes for leaf rust (Lr) and yellow rust (Yr) with different modes of action. The resistance breakdown in 2023 was associated with all-stage (AS) race-specific genes (Lr1, Lr3, Lr10, Lr14a, Lr2a, Lr24, Yr2, Yr4, Yr6, Yr7, Yr9) and adult plant resistance genes (Lr13 race-specific, Lr20, Yr14 race-specific, Lr34 slow rusting), as well as resistance genes carried by wild relatives.

When examining the overall worldwide population of yellow rust using 19 Yr genes, Ali et al. (2017) indicated that virulence to Yr2, Yr6, Yr7, Yr9, and Yr25 was generally in high frequencies and across many regions (>70%). This finding is in accordance with the results of our study, but it has to be pointed out that the virulences to Yr2, Yr6, Yr7, and Yr9 were recorded in Serbia for the first time in 2023, contrary to other European countries. Ali et al. (2017) also reported that virulence to Yr5 and Yr15 were absent in all regions, and virulence to Yr27 was not detected in Europe; virulence to Yr3, Yr4, Yr17, Yr32, YrSp, and YrAmb was absent in West Asia; virulence to Yr10 and Yr24 was absent in North Africa and South Asia; virulence to Yr10, Yr24, and YrSp was absent in Central Asia; and virulence to Yr10, Yr32, and YrSp was absent in East Africa.

Resistance to leaf rust in wheat is currently provided with more than 80 Lr genes and 14 other genes that have not been assigned a new number in the Lr series yet since they have not been subjected to the test of allelism with the known Lr genes (Dakouri et al., 2013). The majority of Lr genes provide race-specific resistance that is expressed with a hypersensitive response (HR) or programmed cell death (Flor, 1946). According to Dakouri et al. (2013), Lr1, Lr3, Lr10, and Lr20 were the most prevalent genes worldwide, but these have also been overcome in addition to Lr9 and Lr28 (Kolmer, 1996). In our study, Lr1, Lr3, Lr10, and Lr20 were present in genotypes that lost resistance to yellow rust in 2023 but also exhibited a susceptibility reaction to leaf rust more in 2023 than in previous years.

Adult plant resistance provided by Lr34 (Lr34/Yr18/Sr57/Pm38/Ltn1), Lr46 (Lr46/Yr29/Sr58/Pm39/Ltn2), and Lr67 (Lr67/Yr46/Sr56/Pm39/Ltn3) expresses pleiotropic effects against three rust species (*Puccinia graminis* f. sp. *tritici*, *Puccinia triticina*, *Puccinia striiformis* f. sp. *tritici*) and powdery mildew (*Blumeria graminis* f. sp. *tritici*) (Lagudah et al., 2006; Hulbert et al., 2007; Kolmer et al., 2008; Herrera-Foessel et al., 2014). Partial or slow resistance to leaf rust provided by Lr34, Lr46, and Lr67 is characterized by a shorter period of sporulation, smaller uredinia size, lower spore density, a longer latent period, and lower infection frequency (Caldwell et al., 1968). The mode of action of Lr34+, Lr46+, and Lr67+ genes does not fit any other previously described disease defense mechanisms (Hulbert et al., 2007), and it was also reported that their effectiveness is dependent on the geographic region (Hulbert et al., 2007). In the study of Dakouri et al. (2013), 79% accessions carring Lr34 had high to moderate levels of resistance ranging from trace level to 35%.

Characterization of APR-related defense responses was usually conducted on transgenic plants in controlled conditions (Risk et al., 2012; Risk et al., 2013), and it was shown that senescence, abiotic stress, and pathogenesis-related (PRs) genes were highly induced in genotypes having the Lr34+ gene. In addition, it was reported that Lr34+ can act synergistically with other leaf rust resistance genes such as Lr12, Lr13, Lr22a, Lr46, Lr67, and Lr68, or novel APR genes (Dyck, 1991; Dyck, 1992; Sawhney, 1992; Kloppers and Pretorius, 1997; Li et al., 2010; Dakouri et al., 2013). However, in our study, Lr34+Lr13 was less effective in providing rust resistance in 2023 than in previous years. Dakouri et al. (2013) reported the possibility of higher levels of rust infections (ranging from 36% to 52%) in genotypes carrying Lr34+, but the reasons for this were only speculated. Sha et al. (2005) reported that DNA methylation can play a role in the expression of APR genes in rice, with a high correlation between the repression of gene expression and hypermethylation but additional analysis of gene expression and methylation patterns is required to test this hypothesis.

According to a report by Kissoudis et al. (2014), 60% of expression changes resulting from combined stressors cannot be predicted based on individual stressor responses. This suggests that the differences between susceptible and resistant plant reactions may be more influenced by variations in the timing and intensity of plant responses to multiple stressors, rather than solely relying on the expression of individual genes (van Loon et al., 2006). Consequently, analyzing the association of morphological and physiological resistance traits conferred by APR genes with abiotic stressors and the expression of abiotic stress genes in field conditions could yield a more detailed genotype profile, facilitating informed breeding decisions to enhance resistance against combined biotic and abiotic stressors.

Nowadays, a lot of effort is being made to solve pest problems using integrated pest management (IPM) with the main goal of minimizing risks to people and the environment. IPM principles are combined to create IPM programs that include six major components: pest identification; monitoring and assessing pest numbers and damage; guidelines for when management action is needed; preventing pest problems; using a combination of biological, cultural, physical/mechanical, and chemical management tools; and, after the action is taken, assessing the effect of pest management. In addition, one of the most important IPM measures is developing pest-resistant genotypes. The breeding programs usually conduct high-throughput genotype screening to test the breeding material for its resistance, and molecular techniques have been constantly improved to map resistance genes. However, despite our increasing understanding of the genome, it may not fully explain the complexity of the phenotype, and identifying complete QTL interactions can be challenging (Velu and Singh, 2013). Our results contribute to all aspects of IPM components, especially those dealing with tracking rust diseases and assessing genotype susceptibility/resistance reactions to rust infection.

## 5 Conclusion

The ability to assess and predict the risk of pathogen development is one of the main goals in plant protection. We indicated that a single genotype could exhibit differences in susceptibility reactions to leaf and yellow rust even in years that are conducive to yellow rust outbreaks. The specificity in genotype reactions to combined abiotic and biotic stressors could directly influence the relationship between rust diseases, leading to the predominance of one pathogen over the other or their coexistence on the same host plant. Monitoring and assessing rust prevalence are important components of IPM, contributing not only to tracking the spread of rust diseases over large areas but also to developing a Decision-Support System for managing fungicide applications. In practice, there are many models for predicting rust occurrence that are based solely on weather data and developed using only a few wheat varieties as models. There is also a lack of information on their long-term efficacy. Therefore, our results indicate that more attention should be focused on multidisciplinary investigations when building models for rust occurrence. Finally, phenotypic plant breeding will always remain an important practice, and our study points out that further investigations should be conducted to identify predictor variables that accurately reflect the response of individual genotypes to combined abiotic and biotic stressors, ensuring more reliable rust control management.

## Data availability statement

The raw data supporting the conclusions of this article will be made available by the authors, without undue reservation.

## Author contributions

RJ: Conceptualization, Funding acquisition, Investigation, Project administration, Resources, Supervision, Writing – review & editing. VŽ: Conceptualization, Formal Analysis, Investigation, Software, Visualization, Writing – original draft.
